# Trichiasis as a clinical manifestation of subconjunctival epithelioid hemangioendothelioma: a rare case report

**DOI:** 10.3389/fonc.2025.1638365

**Published:** 2025-09-04

**Authors:** Mingjue Hu, Jinlin Wang, Sirui Zhou, Yuanyuan Chen, Ting Luo, Juan Guo, Zhanfeng Wang, Yi Sun

**Affiliations:** ^1^ Department of Ophthalmology, The Third People’s Hospital of Chengdu, Chengdu, Sichuan, China; ^2^ Department of Pathology, The Third People’s Hospital of Chengdu, Chengdu, Sichuan, China

**Keywords:** epithelioid hemangioendothelioma, orbit, trichiasis, subconjunctival mass, surgical excision, histopathological diagnosis

## Abstract

Epithelioid hemangioendothelioma(EHE) is a rare, low-grade malignant tumor of vascular endothelial origin, commonly found in the lungs, liver, bones, and soft tissue. However, orbital involvement is uncommon. This study presents a rare case of EHE located in the orbit and confined to the subconjunctival space which has not been previously documented. An elderly male patient presented with a six-month history of discomfort in his left eye, initially diagnosed with lower eyelid trichiasis and scheduled for surgical intervention. A detailed examination identified a purplish, spherical mass beneath the fornix conjunctiva of the left lower eyelid. Computed tomography (CT) imaging of the orbit revealed a well-defined soft tissue mass beneath the left globe, adjacent to the inferior rectus muscle, with no evidence of invasion into the orbital cavity. Ultrasound showed a hypoechoic mass with abundant internal vascularity. Following complete surgical excision, histopathological analysis confirmed the diagnosis of EHE. Postoperative follow-up revealed no signs of recurrence, with full resolution of the trichiasis. No eye movement disorders or visual abnormalities were observed. Despite its generally low malignancy, EHE carries a risk of recurrence and metastasis. Complete excision is crucial to minimize recurrence risk, and long-term follow-up is essential for optimal management.

## Introduction

Epithelioid hemangioendothelioma (EHE) is a rare vascular tumor primarily affecting soft tissues, with most cases found in the lungs, liver, lymph nodes, brain, and bones. Orbital occurrences are notably rare (1, 2). The incidence of EHE is less than 1 in 100,000, with a prevalence of less than 1 in 1,000,000 (1, 3, 4). First described by Weiss and Enzinger in 1982, EHE is characterized histologically and clinically as falling between hemangiomas and angiosarcomas (5). According to the 2020 World Health Organization (WHO) Classification of Tumors of Soft Tissue, EHE is officially categorized as a malignant vascular tumor (6). The clinical presentation of the tumor exhibits variability. In many cases, it is incidentally discovered in asymptomatic patients. Among cases presenting with symptoms, the most common clinical manifestations include pain (40%), a palpable mass (6%-24%), and weight loss (9%) (3). Histologically, EHE is characterized by epithelioid cells embedded within a myxohyaline stroma, the cells display abundant eosinophilic cytoplasm and intracytoplasmic vacuolar structures, which occasionally contain erythrocytes (1, 3). Immunohistochemical result shows positivity for endothelial markers such as CD34, CD31, FLI-1, and ERG, with molecular detection of the WWTR1::CAMTA1 (>90% cases) or the YAP1::TFE3 gene fusion (1). Since its initial description, very few cases of EHE have been reported involving the orbit and/or eyelid. Here, we present the first reported case of EHE confined to the subconjunctival space. At initial examination, the patient presented with lower eyelid entropion and trichiasis without other common orbital tumor symptoms, such as tearing, vision loss, proptosis or displacement, or restricted eye movement. This unusual presentation differs from prior similar cases, potentially due to the tumor’s specific location.

## Case report

**Figure 1:**
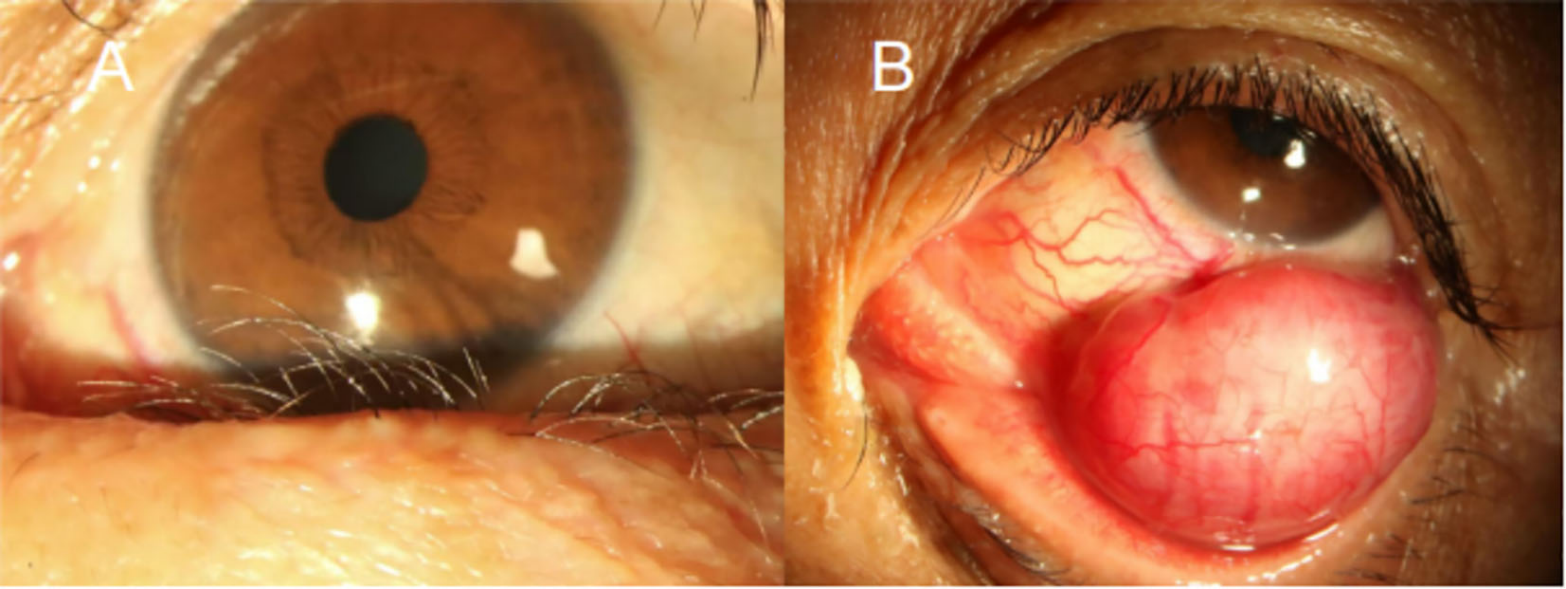
Anterior segment images of the patient’s left eye. **(A)** Medial lower eyelid showing inwardly turned eyelashes (trichiasis). **(B)** Everted lower eyelid revealing a spherical, purple-red sub-conjunctival mass approximately 1.5 cm in diameter.

**Figure 2:**
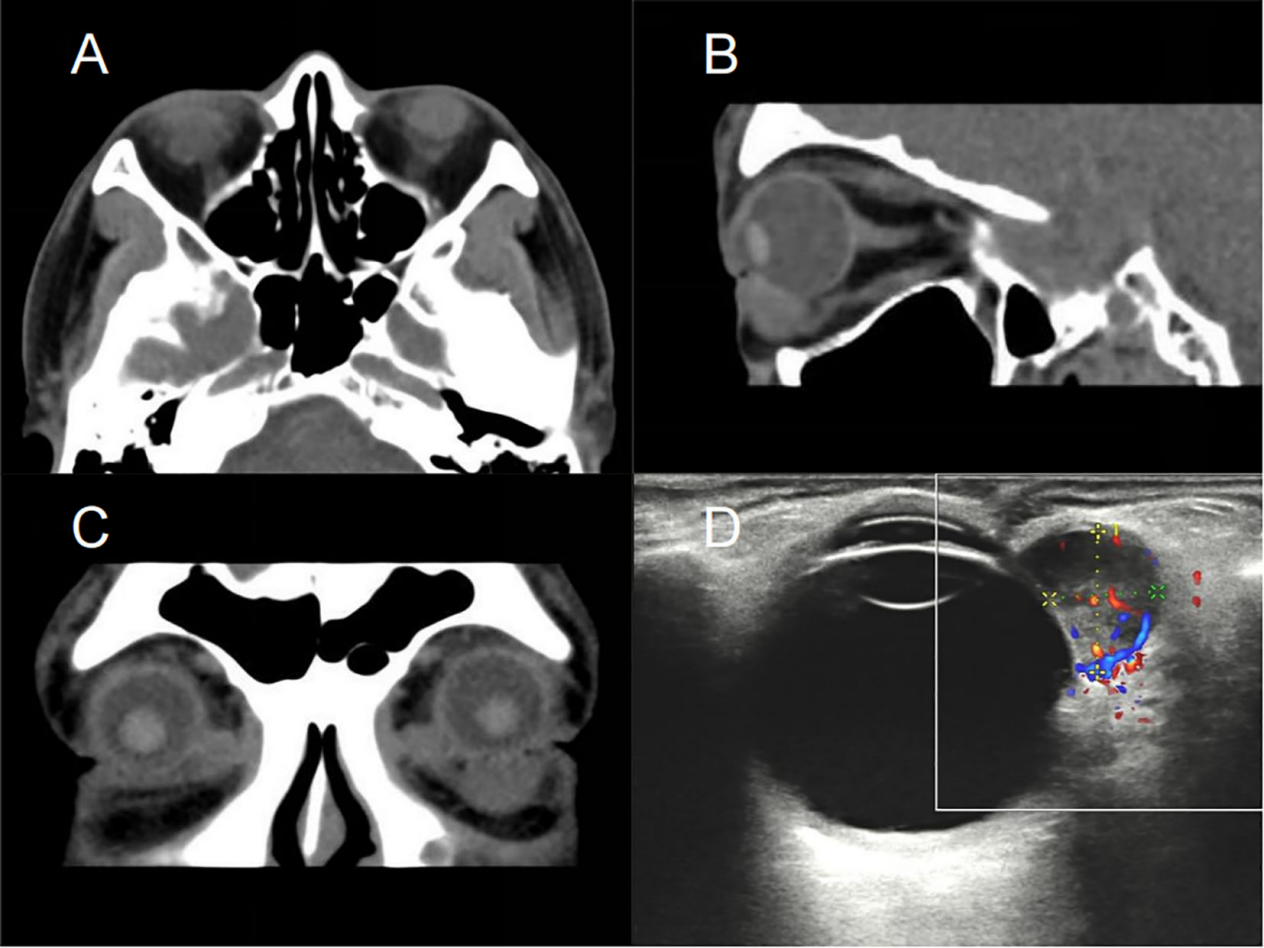
CT scans and ultrasound of the patient’s orbit. Axial **(A)**, sagittal **(B)**, and coronal **(C)** CT images reveal a well-defined, ovoid soft tissue mass located beneath the left globe, with no evidence of invasion into the eye globe or adjacent orbital bones. **(D)** Ultrasound imaging reveals a hypoechoic mass adjacent to the lateral aspect of the left eyeball, measuring approximately 1.50×0.90×0.75 cm. CDFI displayed abundant blood flow signals within the mass, indicating a well-vascularized lesion. CT, Computed Tomography; CDFI, Color Doppler Flow Imaging.

A 59-year-old male presented with a foreign body sensation in the left eye for six months. An initial examination in the outpatient clinic revealed entropion with trichiasis in the left lower eyelid, and surgical correction for the trichiasis was planned. Upon admission, further examination showed visual acuity of 1.0 in both eyes, with normal ocular alignment and eye movements. No significant abnormalities were noted in the anterior and posterior segments of the right eye. The left lower eyelid showed trichiasis (**Figure 1A**) without obvious swelling. On palpation, a firm, poorly mobile, non-tender mass was found in the central region of the left lower eyelid. When the patient looked upward and the lower eyelid was everted, a spherical, purple-red mass approximately 1.5 cm in diameter was exposed in the palpebral fissure (**Figure 1B**), which receded when the eye rotated downward. A more detailed examination of the anterior segment and fundus revealed no other abnormalities. The patient stated no systemic medical or family history. Computed tomography (CT) imaging examination showed a rounded, mass-like soft tissue density shadow below the left eye, adjacent to the inferior rectus muscle, with a clear boundary (**Figures 2A–C**). No abnormalities were found in the eyeball or orbital bones. Ultrasound of the orbit revealed a low-echo mass in the soft tissue outside the left lower eyelid and eyeball, measuring approximately 1.50×0.90×0.75 cm in size, with a well-defined boundary and uniform internal echogenicity. Color Doppler Flow Imaging (CDFI) displayed abundant blood flow signals within the mass (**Figure 2D**). Systemic tumor screening including chest CT and abdominal ultrasound examinations, was normal. Routine blood tests, C-reactive protein levels, and infectious disease screening (including hepatitis B and TORCH) were also unremarkable.

**Figure 3:**
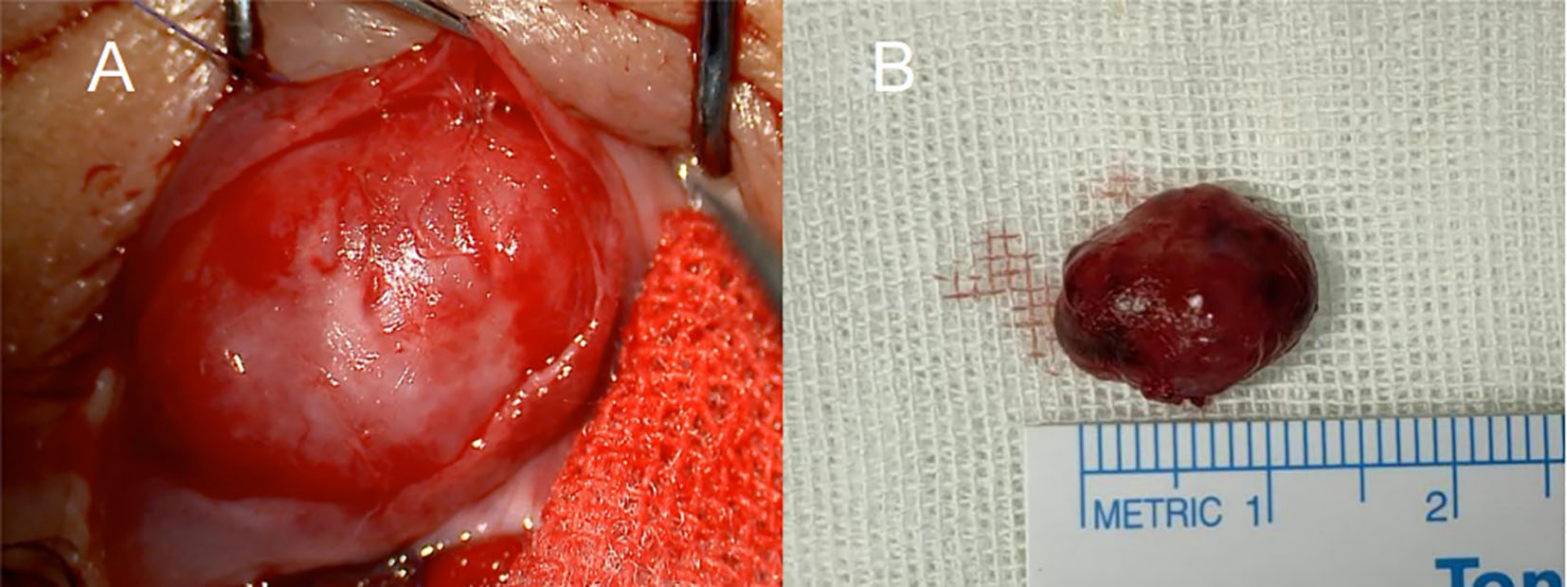
Intraoperative findings of tumor resection. **(A)** The highly vascularized surface of the tumor and surrounding tissue were exposed through an anterior conjunctival incision. **(B)** Image of the intact tumor following surgical resection, measuring approximately 1.5 cm × 1.1 cm × 0.7 cm.

We subsequently performed a conjunctival approach mass excision surgery. Under local infiltration anesthesia, the conjunctiva above the mass was incised, revealing a sarcoma-like mass encapsulated in Tenon’s capsule. After fully exposing the tumor boundary, blunt dissection was performed to separate the tumor mass from the scleral wall. The mass was excised entirely after clamping the vascular pedicle with hemostatic forceps. We found a slight adhesion between the tumor capsule and the inferior rectus muscle tendon sheath, with the mass extending to the orbital septum. The tumor was highly vascularized, with a network of thick, tortuous blood vessels beneath the conjunctiva supplying it (**Figure 3**). The mass was excised intact, measuring approximately 1.5 cm × 1.1 cm × 0.7 cm in size. The inferior rectus muscle was undamaged during the surgery.

**Figure 4:**
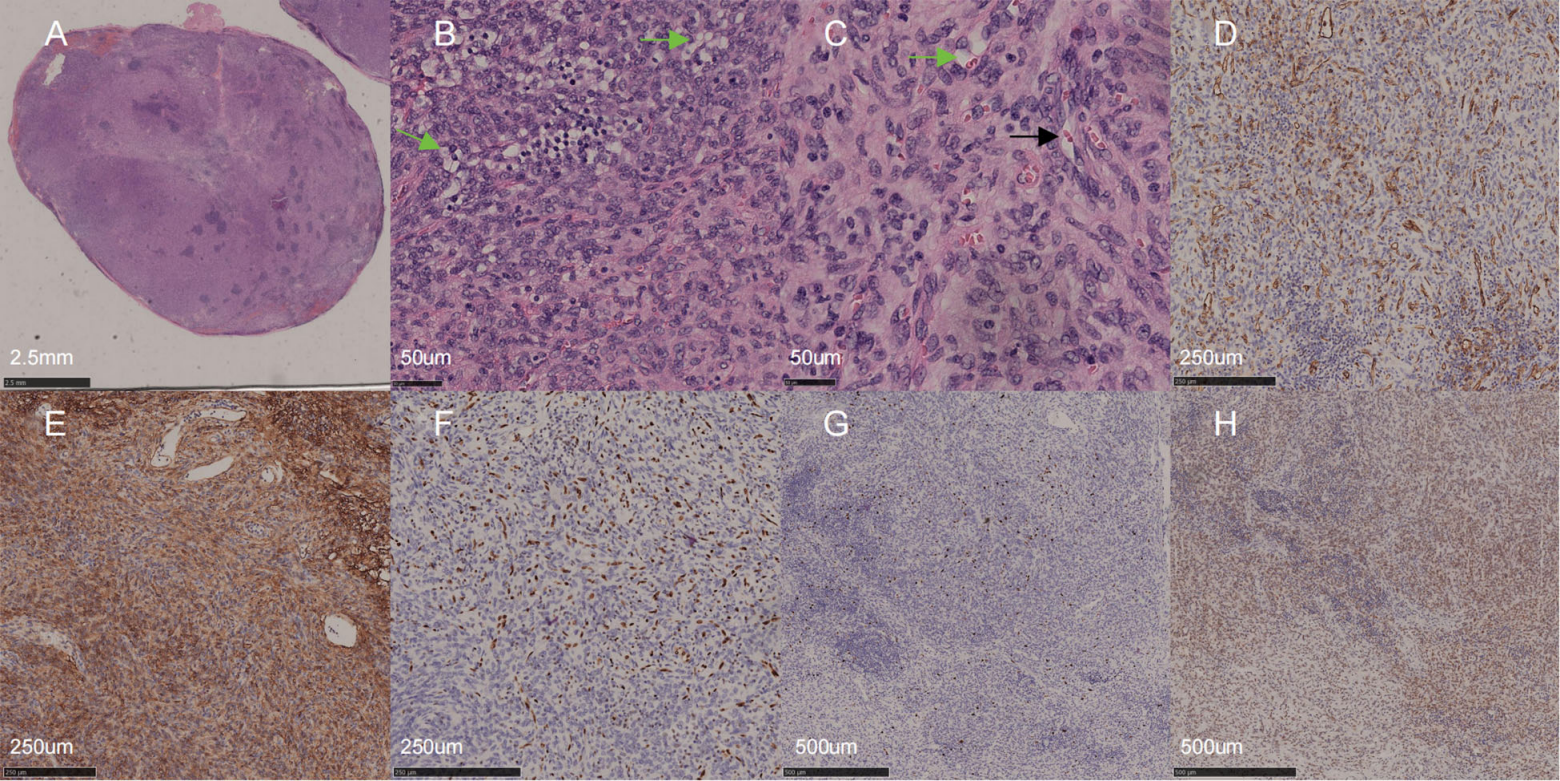
Hematoxylin and eosin staining and immunohistochemistry findings. **(A)** (H&E, scale bar = 2.5mm). Tumor cells with a well-defined boundary are arranged in solid, epithelioid patterns. **(B)** (H&E, scale bar = 50um). The tumor cells exhibit epithelioid morphology with abundant eosinophilic cytoplasm, vesicular nuclei, and no mitotic cells are observed. Intracytoplasmic vacuoles are present (green arrow). Focal but non-dominant angiogenesis is noted. **(C)** (H&E, scale bar =50um). Cytoplasmic vacuoles containing multiple erythrocytes (green arrow) and primitive vascular lumen formation (black arrow) are noted. **(D–H)** Immunohistochemical analysis [Staining intensity: scored as 0 (negative), 1+ (weak), 2+ (moderate), 3+ (strong)]:tumor cell show positivity for vascular endothelial markers, strong CD34 expression (3+, 90% positive cells, E), focal weak CD31 positivity (1+, 20% positive cells, D), and moderate nuclear ERG positivity (2+, 20% positive cells, F), collectively supporting vascular endothelial differentiation. **(G)** Ki-67 proliferation index is approximately 10%. **(H)** TFE3 staining: tumor cells display diffuse and strong positive (3+, >90% positive cells). **(D–F)** scale bar = 250 um. **(G, H)** scale bar =500 um. H&E, Hematoxylin and Eosin Staining; CD34, Cluster of Differentiation 34; CD31, Cluster of Differentiation 31 (Platelet Endothelial Cell Adhesion Molecule-1, PECAM-1); TFE3, Transcription Factor Binding to IGHM Enhancer 3; Ki-67, Proliferation marker protein Ki-67; ERG, ETS-Related Gene Transcription Factor.

Postoperative histopathological examination indicated that the mass had an intact capsule with negative margins and consisted of epithelioid tumor cells arranged in sheets, nests, or cords. The nuclei were vacuolated with indistinct nucleoli, no mitotic cells were observed. Intracytoplasmic vacuoles wrapping erythrocytes were found. Vascular formation was minimal, and focal areas of stromal mucinous degeneration were present (**Figure 4**). Immunohistochemical staining confirmed the diagnosis of EHE, with markers as follows: SMA (−), CD10 (−), S100 (−), CD34 (+), ALK (−), Desmin (+, partial), Ki67 (+, 10%), CD21 (+, FDC network), CD23 (−), CD31 (+), ERG (+, partial), and TFE3 (+). One week and one month postoperatively, the patient’s left lower eyelid entropion was corrected, and he reported no ocular discomfort. No recurrence or metastasis has been observed to date, and we continue to follow up closely with the patient.

## Discussion

EHE is a rare borderline malignant tumor that originates from vascular endothelial cells, typically arising from veins or arteries, especially medium- or large-sized vessels (1). The tumor exhibits diverse presentations, ranging from single-lesion involvement of one organ to multifocal or multi-organ involvement (1). EHE can occur across all age groups and shows no significant gender predilection (7, 8). A comprehensive review of relevant literature reveals only 11 reported cases of primary orbital EHE (**Table 1**), none of which originated in the subconjunctival region. Jamshidian-Tehrani et al. (9) suggested orbital EHE exhibit more aggressive characteristics, including faster growth rates and extension into orbital tissues, compared to EHE in other regions. This may be related to the vascular nature of the tumor, but further evidence is needed to confirm this. In contrast, our case exhibited features more consistent with a benign tumor, likely related to the tumor’s unique location.

**Table 1:** Summary of 12 studies reporting cases of orbital epithelioid hemangioendothelioma.

Case	Age	Sex	Affected Eye	Location	Clinical features	Immunohistochemistry	Distant metastasis	Recurrence	Follow-up	Reference
1	2 months	M	L	Zygomatic and Maxillary Bone	Upward displacement of the eyeball	desmin	No	No	20 months	(26)
2	29 years	M	R	Lateral Orbital Wall, extending from frontal bone into the cranial cavity	Protrusion of the eyeball with diplopia	vimentin, Factor VIII	No	No	30 months	(2)
3	3 years	F	L	Posterior Orbital and Intracranial	Headache, vomiting, eyeball protrusion, and palatal prominence	vimentin, CD34	No	Yes	Died 2.5 months after diagnosis	(7)
4	22 years	F	L	Lacrimal Gland	Swelling on the lateral side of the eyelid	CD31, CD34, EMA	No	No	44 months	(12)
5	13 years	M	R	Near Medial Rectus Muscle	Protrusion of the eyeball with lateral displacement	CD31, CD34	No	No	6 months	(20)
6	55 years	M	R	Upper Eyelid	Enlargement on the medial side of the upper eyelid	CD34	No	No	5 years	(10)
7	30 years	M	L	Retrobulbar	Protrusion of the eyeball with decreased vision	vimentin, CD31, CD34	No	No	2 months	(9)
8	70 years	M	R	Anterior Lateral to the Eyeball	Ptosis with medial displacement of the eyeball	vimentin, CD31, EMA, CD68	No	No	3 months	(11)
9	4 months	M	R	Zygomatic and Maxillary Bone	Medial and upward displacement of the eyeball	CD31, CD34	No	No	12 months	(18)
10	47 years	F	L	Muscle Cone	Decreased vision with eyeball protrusion	CD31, CD34, Factor VIIIa	No	No	36 months	(24)
11	12 years	M	L	Superior to the Eyeball	Ptosis, downward displacement and protrusion of the eyeball with decreased vision		N.R.	N.R.	Died 6 months after surgery	
12	22 years	F	R	the lateral wall of the right orbit	Painful periorbital swelling (right)、diplopia、epiphora	N.R.	No	No	3 months	(25)
13	59 years	M	L	Subconjunctiva	Foreign body sensation, trichiasis	CD31, CD34,TFE3	No	No	1 month	This report

M, male; F, female; R, right eye; L, left eye; N.R, no record.

The etiology of EHE remains unclear, but one of its characteristic features is the presence of gene fusions, including *WWTR1-CAMTA1* and *YAP1-TFE3* (1). Studies indicate that *WWTR1-CAMTA1* and *YAP1-TFE3* account for approximately 90% and 10% of EHE cases, respectively (1, 4), and play a central role in the pathogenesis of EHE. However, the identification of these fusion genes has no prognostic or predictive value, nor does it provide guidance for treatment stratification or medical decision-making; it serves only as an auxiliary tool in molecular testing for confirmation or when the diagnosis is uncertain (3).

In the diagnosis of orbital lesions, CT and MRI imaging can help determine the precise location, extent, and vascular characteristics of the tumor; however, imaging typically cannot provide a definitive diagnosis (10). The histopathological features of EHE include endothelial cell atypia and intratumoral hemorrhage due to ruptured dilated vessels, which may be suggested by imaging findings, as reported by Su et al. (11). However, in this case, the CT scan showed a clearly defined mass with homogeneous internal density, without evidence of fluid or gas-containing structures, fluid stratification, cysts, or cyst-like structures. Furthermore, postoperative pathological examination confirmed it to be a solid mass without intratumoral hemorrhage. It was challenging to determine the vascular characteristics preoperatively based on imaging alone, therefore, the final diagnosis in our case relied on postoperative histomorphological and immunohistochemical findings.

Typical pathological characteristics of EHE include epithelioid tumor cells distributed within a myxoid matrix, arranged in small nests or cords. In hematoxylin and eosin (H&E) staining, the tumor cells usually exhibit eosinophilic cytoplasm with vacuoles, some of which may contain red blood cells (3, 7, 12). Immunohistochemical staining shows positive expression of endothelial markers such as CD31, CD34, ERG, and FLI-1 (1, 13). In this case, H&E staining was consistent with the above features, and immunohistochemical results showed positive expression of CD31, CD34, and ERG, confirming the diagnosis of EHE.

Differential diagnoses of EHE include hemorrhagic cysts, anastomosing hemangioma(AH), benign spindle-cell hemangioma(SCH), epithelioid hemangiomas(EH), and epithelioid angiosarcomas (EA). In our case, cysts were excluded based on the absence of cystic structures, and angiosarcomas were ruled out due to the presence of a low Ki-67 index and the absence of necrosis (7, 10, 11). AH features interconnected vascular channels lined with hobnail endothelial cells, with positive staining for CD31, CD34, ERG, and SMA, expressed in pericytes (14, 15). SCH is characterized by cavernous vascular spaces and spindle cell areas, expresses endothelial markers and SMA (16). However, these morphological features were not observed in our case, and SMA expression was negative, thereby excluding both SCH and AH. EH is composed of capillary-like small vessels. In contrast, vascular formations were sparsely found in the present case, making EH an unlikely diagnosis (17). Based on the morphological features and immunohistochemical findings, we ruled out the above differential diagnoses, supporting a diagnosis of EHE. The patient’s Ki-67 index was 10% in this case, indicating low tumor proliferative activity, suggesting a low risk of recurrence and metastasis.

The treatment strategy for EHE varies depending on the tumor location and malignancy potential (7, 8). Given the rarity of EHE, treatment guidelines are not well established, and the primary approach currently is complete surgical resection (9, 18). Due to the typically rich vascular supply of EHE, some studies suggest preoperative embolization to minimize intraoperative bleeding risk (8). However, Jamshidian-Tehrani et al. (9) reported a case in which preoperative embolization led to occlusion of the central retinal artery and vein; while the patient’s visual outcome was ultimately favorable, embolization of the central retinal artery or cavernous sinus carries potential risks. Chalkiadaki et al. proposed an innovative method involving intralesional injection of N-butyl-2-cyanoacrylate and 60% Lipiodol, which allowed complete tumor resection with minimal blood loss and did not interfere with subsequent pathological evaluation (19). For cases without visible vascular supply or vascular malformations on imaging, pre- or intraoperative tumor embolization may still be considered, though its efficacy and safety require further investigation. In this case, blunt dissection and full exposure of the tumor were performed, with vascular control achieved using hemostatic forceps before resection, resulting in minimal intraoperative bleeding.

While chemotherapy and radiotherapy show limited effectiveness in EHE, they may serve as adjunct therapies in cases of disease progression or metastasis (11, 20). Chemotherapy for EHE is typically adapted from that for soft tissue sarcomas. Cytotoxic chemotherapy provides only palliative benefit without improving survival (21). Targeted therapies such as pazopanib and sirolimus have shown modest efficacy. Further studies are warranted to explore tumor angiogenesis and molecular signaling pathways as therapeutic strategies (1). Adjuvant radiotherapy at doses of 60 Gy is recommended for high-risk cases. In patients with multifocal or metastatic disease, radiation doses vary from 30 to 60 Gy depending on symptoms and tumor burden (3). However, large-scale studies to guide optimal radiation dosing are lacking. Although EBRT may provide symptomatic relief, it offers limited long-term control (22). Immunotherapy and plaque brachytherapy lack substantial evidence for EHE (1, 23). In this case, complete surgical excision was achieved with histopathologically confirmed negative margins, and systematic evaluation revealed no evidence of metastasis. Therefore, adjuvant systemic therapy was considered unnecessary.

The prognosis of EHE remains uncertain, as it not only has the potential for local recurrence but may also lead to distant metastasis (10). Predicting the risk of metastasis in orbital EHE is particularly challenging, as there are no documented cases of distant metastasis in the current literature for orbital EHE (**Table 1**). Nevertheless, due to the unpredictable nature of the disease, close follow-up is essential for confirmed cases. An expert consensus (3) recommends that patients with EHE undergo MRI of the primary site and whole-body CT screening every six months during the first four to five years postoperatively. If no evidence of progression is detected during this period, annual examinations may be sufficient.

This report has several limitations. Firstly, the follow-up duration is less than five years, and we can only acknowledge the patient’s current status through telephone communication; therefore, no postoperative imaging data are available for the patient. Second, this study reports only one patient. To accurately evaluate the prognosis of EHE multicenter, large-sample studies with long-term follow-up and randomized controlled designs are necessary. Additionally, due to limitations in our laboratory testing capabilities and the patient’s financial constraints, molecular testing could not be performed, which limited a more comprehensive analysis of the relationship between tumor molecular characteristics and clinical prognosis. Future investigations should focus on addressing these issues to provide more reliable, evidence-based guidance for the precision diagnosis and treatment of EHE.

## Conclusion

To our knowledge, this is the first reported case of EHE localized exclusively in the subconjunctival region. This case underscores the importance of cytomorphology and immunohistochemistry as critical diagnostic tools for EHE. Complete surgical resection combined with close monitoring remains the cornerstone of effective management. Through a review of relevant literature, this study highlights the clinicopathological characteristics of EHE, providing valuable guidance for its diagnosis, treatment, and follow-up. Due to the rarity and sporadic occurrence of orbital EHE, comprehensive analysis of its epidemiology, clinical features, and prognosis remains challenging, emphasizing the need for further case accumulation to deepen understanding.

## Data Availability

The original contributions presented in the study are included in the article/supplementary material. Further inquiries can be directed to the corresponding author.
